# Dietary pulse prebiotic fibre intake in a rat obese pregnancy model alters maternal caecal microbiome and protects against steatosis in newly weaned offspring

**DOI:** 10.1017/jns.2026.10103

**Published:** 2026-05-26

**Authors:** Simran Utreja, Gabriella A. Andreani, Saleh Mahmood, Mulchand S. Patel, Michael J. Buck, Todd C. Rideout

**Affiliations:** 1 Department of Biochemistry, State University of New York at Buffalo, USA; 2 Department of Exercise and Nutrition Sciences, State University of New York at Buffalo, USA

**Keywords:** Dietary pulse, fibre, maternal obesity, microbiome, pregnancy, short-chain fatty acids

## Abstract

We assessed if supplementation of an obese-inducing diet with yellow pea fibre throughout pre-pregnancy (PP), gestation, and lactation could influence maternal gut microbiome composition and improve metabolic health and liver steatosis in newly weaned rat male and female offspring. Forty female Sprague-Dawley rats were fed a low (**CON**) or high (**HC**) calorie diet for a 6-week PP period. At the end of PP, HC animals were randomly assigned to either remain on the HC diet or the HC diet with yellow pea fibre (**HC + FBR**) for an additional 4-weeks prior to mating and throughout gestation and lactation. At the end of lactation, caecal microbiome profile was evaluated in mothers with shotgun metagenomic sequencing, and newly weaned male and female pups were assessed for serum biochemistry and hepatic fat outcomes. Maternal obesity reduced the beta-diversity of the maternal microbiome and lowered total caecal short-chain fatty acid (SCFA) concentration. HC + FBR consumption increased caecal SCFA concentration and differentially altered the maternal caecal microbiome profile of several species that have been linked with hepatic steatosis including Bifidobacterium pseudolongum, Porphyromonas gingivalis, and several Provetella species. Newly weaned offspring from HC mothers exhibited hepatic steatosis; however, male and female pups from HC + FBR mothers demonstrated normalised liver lipid concentrations (cholesterol and triglyceride) and an increase in caecal acetate and propionate concentrations. Findings suggest that maternal obesity enhances the risk of liver steatosis in offspring and that maternal dietary fibre supplementation may have a protective influence that is partly mediated through changes in the caecal microbiome profile and activity.

## Graphical abstract

**Figure:**
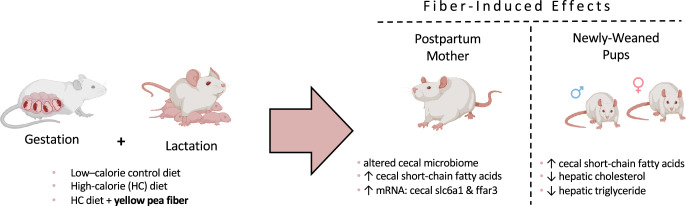

## Introduction

Recent research has emphasised the importance of early-life factors that may contribute to the alarming rise in childhood obesity and associated metabolic diseases such as metabolic dysfunction-associated steatotic liver disease (MASLD).^(1)^ Both human^(2)^ and animal-model^(3)^ studies suggest an increased risk of MASLD in offspring born to mothers with obesity. Maternal obesity strongly influences the maternal microbiome, which in turn may programme offspring metabolism towards an increased predisposition to liver steatosis.^(4)^ Maternal pre-pregnancy body mass index and gestational weight gain (GWG) are associated with gut microbiome changes in mothers, including a decline in bacterial diversity and richness.^(^ ^5^ ^–^ ^8^ ^)^ This is concerning as both animal^(9,10)^ and human^(11,12)^ studies suggest that an obesogenic maternal microbiome can be transferred to offspring through *in utero* fetal exposure^(13,14)^ and during early infancy through milk consumption.^(15,16)^ Accordingly, results of a previous systematic review reported that obese pregnancies were associated with significant changes to the gut microbiome of both mothers and infants, including increases in *Bacteroidetes*, *Firmicutes* and decreases in *Bifidobacteria.* ^(17)^

As diet is a critical environmental factor affecting gut microbiome composition and diversity,^(18)^ optimal maternal nutrition before, during, and after pregnancy is instrumental in ensuring the early-life colonisation of a healthy microbiome in offspring. Previous human studies have reported that the gut microbiome profile in neonates associates with maternal nutrient intake during pregnancy, with a particular influence of dietary fat,^(19)^ dietary fibre^(20)^ and specific food groups including dairy,^(21)^ fruits and vegetables^(22)^. Unfortunately, perinatal maternal nutrition is typically suboptimal, with excess intake of energy, sugar, and saturated fat and nutrient gaps in many micronutrients^(23–26)^ and dietary fibre.^(27)^ Thus, improving maternal diet quality would be expected to not only benefit maternal health but also limit the transgenerational cycle of obesity.

Although widely under consumed in the U.S.,^(28)^ dietary pulses (i.e., dry beans, peas, and lentils) have an outstanding nutritional profile and have been shown to reduce inflammation,^(29,30)^ improve glycaemic control,^(31–34)^ reduce blood lipids^(35,36)^, and support weight reduction.^(30,37–39)^ Additionally, bioactive fractions of pulses, including soluble and insoluble fibre and protein components, have been demonstrated to shift microbiome populations toward commensal, health-promoting species and increase SCFA production.^(40–45)^ Unfortunately, whether maternal pulse crop consumption during pregnancy can improve maternal gut microbiome and subsequently influence offspring health has rarely been investigated. Thus, using an obese Sprague Dawley rat pregnancy model, we sought to determine if supplementation of an obese-inducing diet with yellow pea fibre throughout pre-pregnancy, gestation, and lactation could influence maternal gut microbiome composition and improve metabolic health and liver steatosis in newly weaned male and female offspring.

## Materials and methods

### Animal and diets

Experimental animals were cared for in accordance with the guidelines established by the Institutional Animal Care and Use Committee, and all procedures were reviewed and approved by the Animal Care Committee at the University at Buffalo.

**Figure 1.**
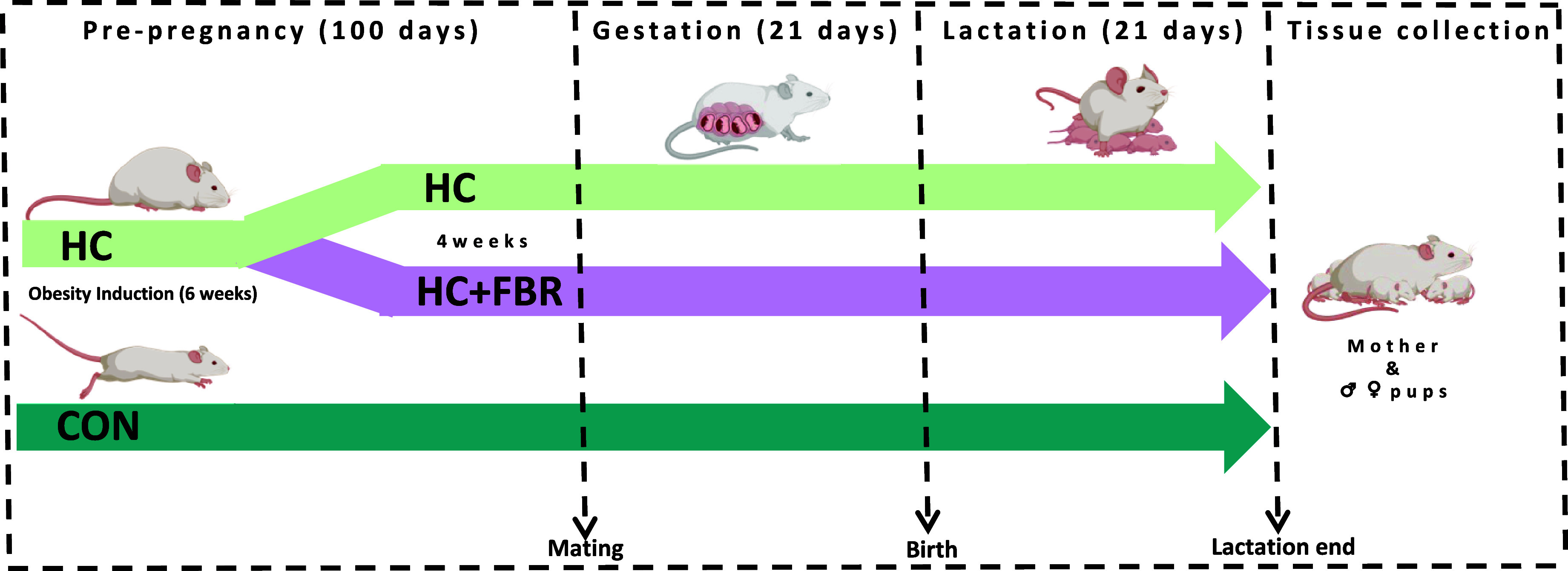
Experimental design.

The experimental design is presented in Figure 1. Forty newly weaned [postnatal day (PND) 21] female Sprague-Dawley rats (Charles River, obese prone, Crl:OP-CD) were housed in the Laboratory Animal Facility at the University at Buffalo under controlled environmental conditions (12 h light:12 h dark, 20°C, and 50% humidity). All animals had free access to food and water throughout the experiment. During the initial 6 weeks of pre-pregnancy, rats were randomised to receive either a low-calorie control diet (**CON**; *n* = 12; total energy 3.8 kcal/g; % energy from fat, 10; protein, 20; and available carbohydrate, 70) (Research Diets, D12450K) or a high-caloric obesity-inducing diet (**HC**; *n* = 28; total energy 4.7 kcal/g; % energy from fat, 45.2; protein, 20.1; and available carbohydrate, 34.7) (Research Diets, D12451) (Table 1). Following this 6-week period, ‘obese’ HC animals (defined as having body weight ≥20% vs. CON) were then randomised to either remain on the HC diet (*n* = 14) or provided the HC diet supplemented with a yellow pea fibre-enriched fraction (**HC + FBR**, *n* = 14, total energy 4.3 kcal/g; % energy from fat, 45.1; protein, 19.8; and available carbohydrate, 34.1) for an additional 4 weeks prior to mating (Table 1). Macronutrient and fibre content of the pea fibre supplement (25% Roquette® Pea Fiber I 50m) was assessed by third-party analyses (Anresco Laboratories, San Francisco, Ca, USA) and reported to have 1.89 kcal/g energy, 7.65% protein, 0.48% fat, 83.0% total carbohydrate, and 48.44% dietary fibre (43.99% insoluble and 4.45% soluble). The HC and HC + FBR diets were designed to be macronutrient-matched (∼20% protein, ∼35% protein, and 45% fat). At end of the pre-pregnancy period (a total of 10 weeks) the rats were bred with lean CON-fed male breeders to establish a timed pregnancy,^(46)^ confirmed by the presence of a vaginal plug. Maternal body weights and food intake were collected weekly throughout gestation. Following delivery, litter size and weights were recorded, and the litters were adjusted to 8 pups per dam (4 males and 4 females where possible) within 24 hours of birth. Mothers continued on their respective test diets throughout lactation. Weekly caloric intake (mothers) and body weights (mothers and litters) were recorded. At the end of lactation, mothers and pups were placed under isoflurane anaesthesia for collection of blood for serum separation and liver tissue that was flash frozen in liquid nitrogen. Additionally, maternal caecal tissue and luminal contents of the caecum were collected and flash frozen in liquid nitrogen.

**Table 1.** Experimental diet formulation^1^

	Diets (% composition)		
Ingredient (%)	CON	HC	HC + FBR
Casein	19.0	23.3	19.0
Corn Starch	52.1	8.5	9.4
Maltodextrin	14.2	11.6	9.0
Sucrose	0.4	20.6	9.7
Cellulose	4.7	5.8	0.0
Lard	1.9	20.7	18.5
Soybean oil	2.4	2.9	2.9
Yellow pea fibre	–	–	25.0
Other^1^	5.3	6.5	6.5
Nutrient profile, % energy			
Protein	20.0	20.1	19.8
Available carbohydrate	70.0	34.7	34.1
Fat	10.0	45.2	45.1
Calories (kcal/g)	3.8	4.7	4.3
Total dietary fibre (g)	4.7	5.8	12.1

1 Including mineral mix, vitamin mix, L-cystine, and choline bitrate; CON, low calorie control diet; HC, high calorie Western diet; HC + FBR, HC diet supplemented with yellow pea fibre. Third party nutrient analyses of the yellow pea fibre extract: total energy, 1.89 kcal/g; % fat, 0.48; % protein, 7.6; % carbohydrate, 82.5.

### Blood biochemistry

Serum analyses included glucose by colorimetric detection (Invitrogen, EIAGLUC), insulin by ELISA (Millipore, EZRMI-13K), cholesterol (TC, LDL/VLDL-C, and HDL-C) by enzymatic analysis (BioAssay, EHDL-100), and triglyceride (TG) by enzyme assay (Zenbio, STG-1-NC).

### Caecal short-chain fatty acids

Short-chain fatty acids (SCFA), including acetate (C2), propionate (C3), and butyrate (C4), were extracted according to previous procedures.^(^ ^47^ ^)^ Briefly, caecal samples (100 mg) were spiked with 100 mM caproic acid (internal standard) for quantification and acidified with sulphuric acid to transform the SCFAs into their undissociated form, thus increasing their hydrophobicity and their volatility.^(48)^ Following motorised homogenisation (1 min @ 20 rpm), the samples were extracted three times with diethyl ether, centrifuged at 2800 x g at 4 °C for 10 minutes. Following centrifugation, the upper organic phase was collected and transferred to a labelled vial. Lipid fractions were analysed on a Shimadzu GC-17A gas chromatograph fitted with a flame ionisation detector using a Restek™ Stabilwax™-DA Capillary Column.

### Hepatic lipids

Hepatic TG was extracted from frozen tissue and analysed with a commercial kit (Zenbio, STG-1-NC) according to previously published work.^(49)^ Hepatic cholesterol was extracted from frozen tissue and analysed on a Shimadzu GC-17A gas chromatograph fitted with a flame ionisation detector using a Restek™ Stabilwax™-DA Capillary Column.^(50)^

### Caecal mRNA extraction and real-time RT-PCR: mRNA expression

Total RNA was isolated from caecal contents (∼100 mg) using RNeasy mini-kit (Qiagen, #74104). RNA concentration and integrity was determined with spectrophotometry (260 nm). RNA preparation and real-time RT-PCR were conducted using a one-step QuantiFast SYBR Green RT-PCR kit (Qiagen, # 204154) with a Biorad CFX96 Touch real-time PCR system. Gene expression was analysed using the 2(-delta delta *C*t) method^(51)^ using *β*-actin as the housekeeping gene. Validated primer sets (QuantiTect Primer Assays, Qiagen) were used for the following genes: *β*-actin (*Actb, GeneGlobe ID: QT00193473*), solute carrier family 5 member 8 (slc5a8, QT00546483), solute carrier family 6 member 1 (slc6a1, QT00182343), free fatty acid receptor 2 (ffar2, QT00384860), and free fatty acid receptor 3 (ffar3, QT01299144).

### Caecal microbiome

The total DNA from caecal contents was extracted using the QIAamp PowerFecal Pro DNA procedure, with the addition of bead beating to maximise yield. In brief, microbiome DNA was isolated from 250 mg of caecum contents using a Bead-Beater (Bio spec) for homogenisation. Bead-beating was performed three times for 1 minute, with the tubes kept on ice for 1 minute between each round. Homogenised samples were then processed following the QIAamp PowerFecal procedure. Isolated DNA was then kept at −20°C until further processing. Shotgun sequencing libraries were generated using NEB Ultra FS II DNA Library Prep (E7805S) for large fragment sizes (>550bp). Pooling and 150 bp paired-end sequencing were done at the UB Genomics and Bioinformatics core using an Illumina NextSeq. Next-generation sequencing (NGS) reads were quality-filtered using Kneaddata with default Trimmomatic parameters.^(52)^ Human and Rat DNA were also removed from the datasets. Taxonomic classification was performed using Kraken2, which was used to align the quality-filtered reads to the Standard database containing bacterial, archaeal, and viral genomes. Abundances were then estimated with Bracken V2.5.^(53,54)^ OTU abundances were loaded into MicrobiomeAnalyst under Marker Data Profiling^(55)^ for further analysis. The dataset was then filtered to remove OTUs with low variance (10%) across the dataset and CLR transformed. For alpha diversity, all groups were compared with ANOVA and pairwise comparisons by Welch’s *T*-test. Beta diversity was performed by PCoA with the Bray-Curtis distance. Linear Discriminant Analysis Effect Size (LEfSe) analysis was performed on TSS-normalised data with FDR 0.01 and LDA >2.5.

### Statistics

Statistical analyses for metabolic assessments were conducted using SPSS 16 (SPSS Inc., Chicago, IL). Using preliminary data demonstrating increased hepatic steatosis (primary outcome) in offspring from diet-induced obese rat mothers, we estimated a required sample size of 6–8 mothers/group (effect size, 1.35; power, 0.85; *p* < 0.05). Data were checked for normality using the Shapiro–Wilk test. Animal treatment groups were blinded to the technicians conducting the laboratory analysis. The trajectory of maternal body weights was analysed across phases (pre-pregnancy, gestation, and lactation) with repeated measures analysis. Maternal metabolic outcomes were measured with a one-way ANOVA with a least significant difference (LSD) post-hoc test to test for pre-planned comparisons. Litters from each dam were considered as a single observation. For offspring outcomes, main effects of maternal diet exposure (CON, HC, and HC + FBR) and sex (male and female from same maternal exposure), and interaction-related effects were analysed by two-way ANOVA. If a significant main effect or interaction was detected, a one-way ANOVA with an LSD post-hoc test was conducted to assess pre-planned comparisons. Data are presented as means ± SE for maternal growth and box and whisker plots (min to max, where the centre line represents the median) for all other outcomes. Differences were considered significant at *p* < 0.05.

## Results

### Maternal outcomes

#### Pregnancy outcomes

Although time to pregnancy (measured in days) was similar between groups (*p* > 0.05), reproductive success, defined as mothers who gave birth without subsequent infanticide of 1 or more pups, was reduced in HC dams (47.1 vs. 91.7% in CON) and rescued in the HC + FBR group (88.9%). Litter size (# pups) and weight at birth were similar (*p* > 0.05) between groups (Table 2).

**Table 2.** Maternal pregnancy, growth, and metabolic outcomes.^1^

Outcome	CON	HC	HC + FBR
**Pregnancy outcomes**			
Time to pregnancy (days)	2.2 ± 0.4^a^	2.3 ± 0.3^a^	1.8 ± 0.5^a^
Reproductive success (%)^2^	91.7	47.1	88.9
Litter size (# pups)	13.8 ± 0.7^a^	14.3 ± 0.5^a^	13.5 ± 0.8^a^
Litter weight (g)	91.9 ± 1.5^a^	87.4 ± 4.7^a^	92.2 ± 4.9^a^
**Body weight**			
End of pre-pregnancy (g)	289.0 ± 5.0^a^	347.9 ± 6.3^b^	351.8 ± 9.0^b^
Gestational weight gain (g)	139.7 ± 7.4^a^	142.5 ± 6.4^a^	148.6 ± 9.5^a^
End of lactation (g)	331.6 ± 11.6^a^	345.9 ± 9.6^a^	358.6 ± 10.4^a^
**Serum biochemistry (end of lactation)**			
Glucose (mg/dL)	84.6 ± 14.3^a^	102.3 ± 18.0^a^	82.9 ± 3.2^a^
Insulin (µIU/mL)	8.9 ± 0.26^a^	7.9 ± 0.33^b^	7.7 ± 0.15^b^
Glucose:Insulin	9.2 ± 1.4^a^	12.1 ± 1.7^a^	8.8 ± 1.2^a^
Total-cholesterol (mg/dL)	93.6 ± 7.6^a^	73.2 ± 3.0^a^	79.1 ± 8.9^a^
HDL-cholesterol (mg/dL)	71.1 ± 6.6^a^	46.4 ± 4.7^b^	49.3 ± 4.4^b^
LDL/VLDL-cholesterol (mg/dL)	9.4 ± 1.8^b^	5.9 ± 1.4^b^	14.4 ± 3.2^b^
Total-C:HDL-C ratio	1.4 ± 0.1^a^	1.5 ± 0.1^b^	1.6 ± 0.1^b^
Triglycerides (mg/dL)	35.1 ± 5.9^a^	33.4 ± 3.0^b^	31.7 ± 3.9^b^

1 ab, groups not sharing a letter are significantly different at *p* < 0.05; *n* = 6–8 animals per group.

2 Calculated as mothers who gave birth without subsequent infanticide.

#### Growth and caloric intake

Measured across all phases (pre-pregnancy, gestation, and lactation), HC and HC + FBR mothers demonstrated increased (*p* < 0.05) body weight vs. CON mothers; however, they did not differ (*p* > 0.05) from each other [*F*(1,19) = 10.80, (*p* = 0.001)]. A similar influence of maternal diet was observed at the end of pre-pregnancy, with the fibre intervention offering no protection (*p* > 0.05) against maternal HC-induced weight gain (Table 2). No group differences (*p* > 0.05) were noted in gestational weight gain or body weight at the end of lactation (Table 2). Cumulative caloric intake (kcal/day) in mothers measured across all phases tended (*p* = 0.10) to be higher in HC (98.7 ± 1.2) vs. CON (90.3 ± 2.9) and was higher (*p* < 0.05) in HC + FBR mothers (107.3 ± 2.4) compared to both other groups.

#### Serum biochemistry

No difference in blood glucose was observed between groups; however, both the HC and HC + FBR groups showed a comparable reduction (*p* < 0.05) in blood insulin vs. CON mothers. However, no change in the glucose:insulin ratio was observed between groups (Table 2). Although serum total and LDL/VLDL cholesterol was similar between groups, HDL-C was reduced in both the HC and HC + FBR groups compared to CON mothers (Table 2). The TC:HDL-C ratio was increased in both the HC and HC + FBR mothers compared with CON. No difference was observed in serum TG between groups.

#### Caecal microbiome

**Figure 2.**
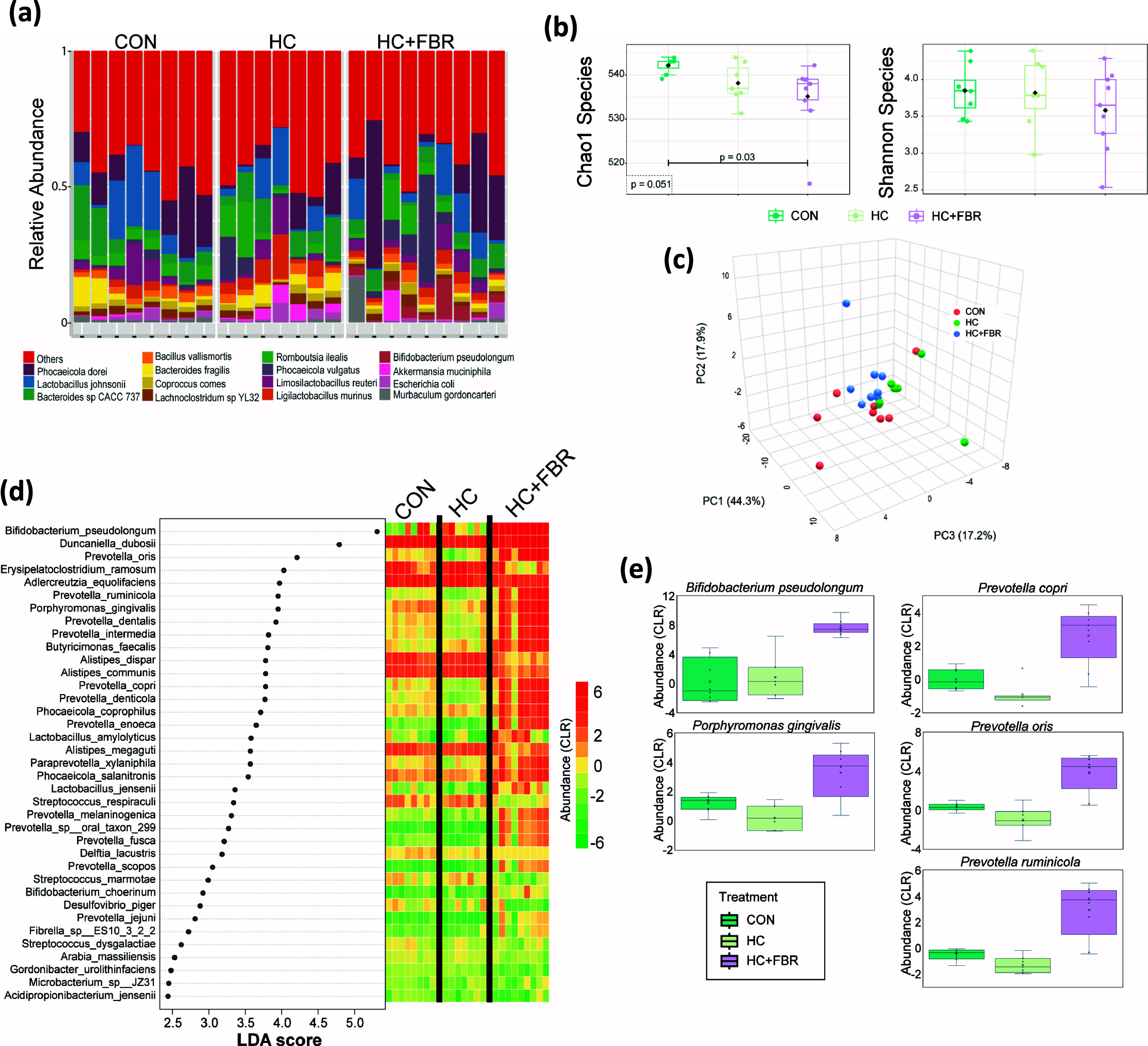
Gut microbiome analysis of maternal dietary intervention. **(a)** Relative abundance at the species level for gut bacteria across CON (control), HC (high calorie), HC + FBR (high calorie with yellow pea fibre); **(b)** Chao1 and Shannon alpha diversity at the species level. **(c)** Beta analysis; **(d)** LEfSe analysis with LDA effect size. The abundance (CLR) of enriched tax is shown for each sample in the heatmap; **(e)** Abundance of differential bacterial tax.

Shotgun sequencing of the caecal microbiome identified 607 unique bacterial taxa at the species level. The majority of bacteria belong to the Bacteroidaceae and Lactobacilaceae families. At the species level Phocaeicola donei and *Lactobacillus johnsonii* were the most abundant (Figure 2a). There was a modest difference in Chao1 α diversity across groups (*p* = 0.051) and a significant drop in diversity comparing HC + FBR to CON (*p* = 0.03) (Figure 2b). For Shannon diversity there was no significant differences between the groups. PERMANOVA analysis showed a significant difference in beta diversity across groups (R^2^ = 0.26, *p* = 0.01) indicating divergent microbial communities (Figure 2c).

To identify the bacterial species that were significantly different between the groups and have a meaningful effect size, we performed Linear Discriminant Analysis Effect Size (LEfSe)^(56)^. LEfSe analysis identified 37 significant bacterial taxa (FDR < 0.1) with an LDA score>2.5 (Figure 2d). The majority of the LEfSe taxa were enriched in HC + FBR when compared to both the control and HC diets (Figure 2e).

#### Caecal short-chain fatty acids

**Figure 3.**
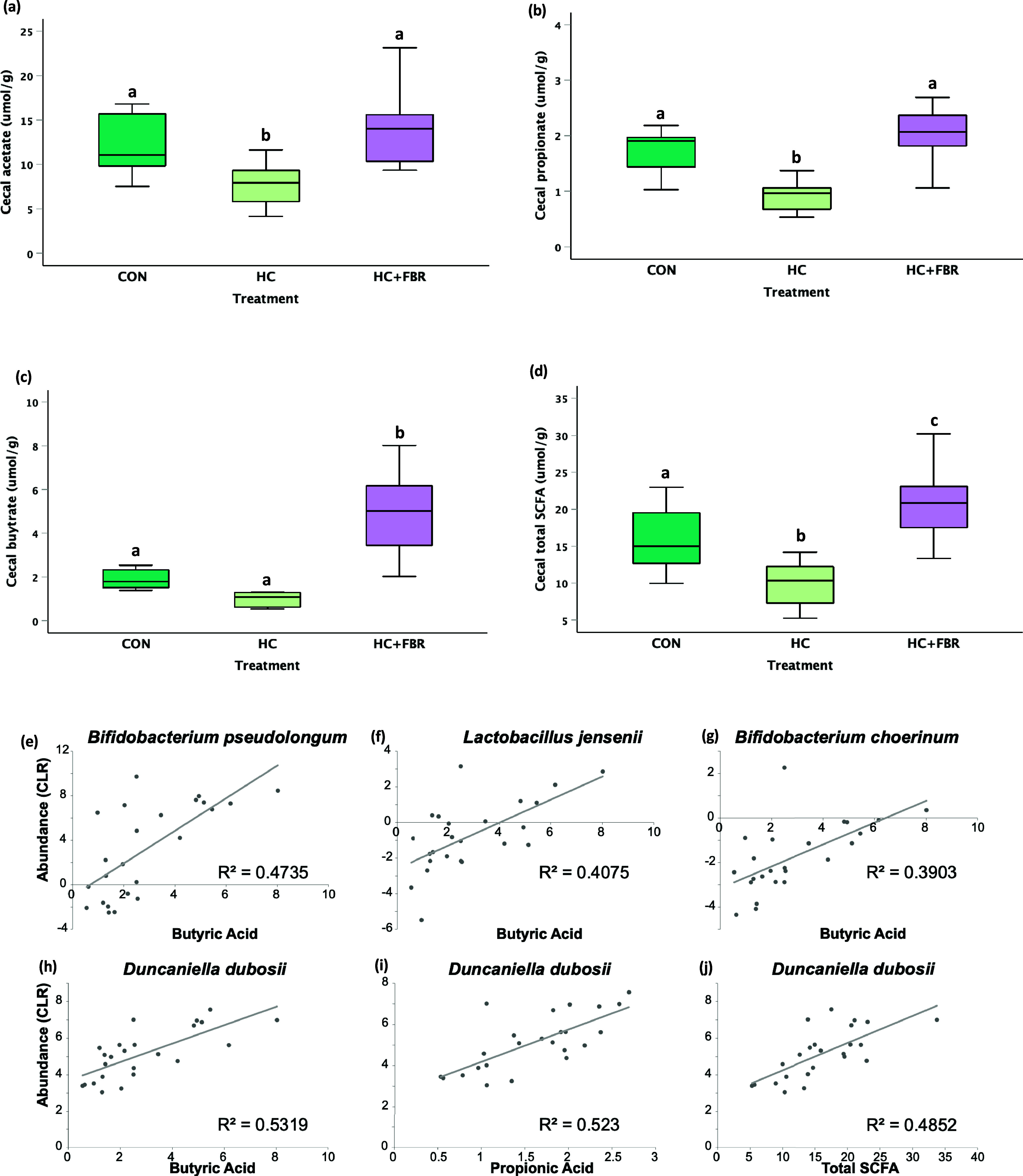
**(a)** Maternal short-chain fatty acid (SCFA) concentration (µmol/g) in luminal caecal contents on lactation day 21 including **(a)** Acetate, (**b**) Propionate; (**c**) Butyrate; and (**d**) Total caecal SCFA. (**e–j**) Depict pearson correlations between caecal SCFA and differentially expressed bacteria at the species level. SCFA data are presented as box and whisker plots, min to max, where the centre line represents the median. Data analysed using a one-way ANOVA with an LSD post-hoc multiple comparison test. **abc**, groups not sharing a letter are significantly different at *p* < 0.05; *n* = 6–8 animals per group.

The concentration of caecal acetate and propionate were lower (*p* < 0.05) in HC mothers compared with the CON and HC + FBR groups (Fig. 3ab). Caecal butyrate was higher (*p* < 0.05) in HC + FBR mothers compared with the CON and HC groups (Figure 3c). The concentration of total caecal SCFA (sum of acetate, propionate, and butyrate) was lower (*p* < 0.05) in HC mothers (vs. CON) but higher (*p* < 0.05) in the HC + FBR group (vs. CON and HC) (Figure 3d). The concentrations in SCFA were compare by correlation to abundance of the 37 significant taxa (Figure 3e). Six correlations are significant after correcting for multiple testing (*p* < 0.05).

#### Caecal gene expression

**Figure 4.**
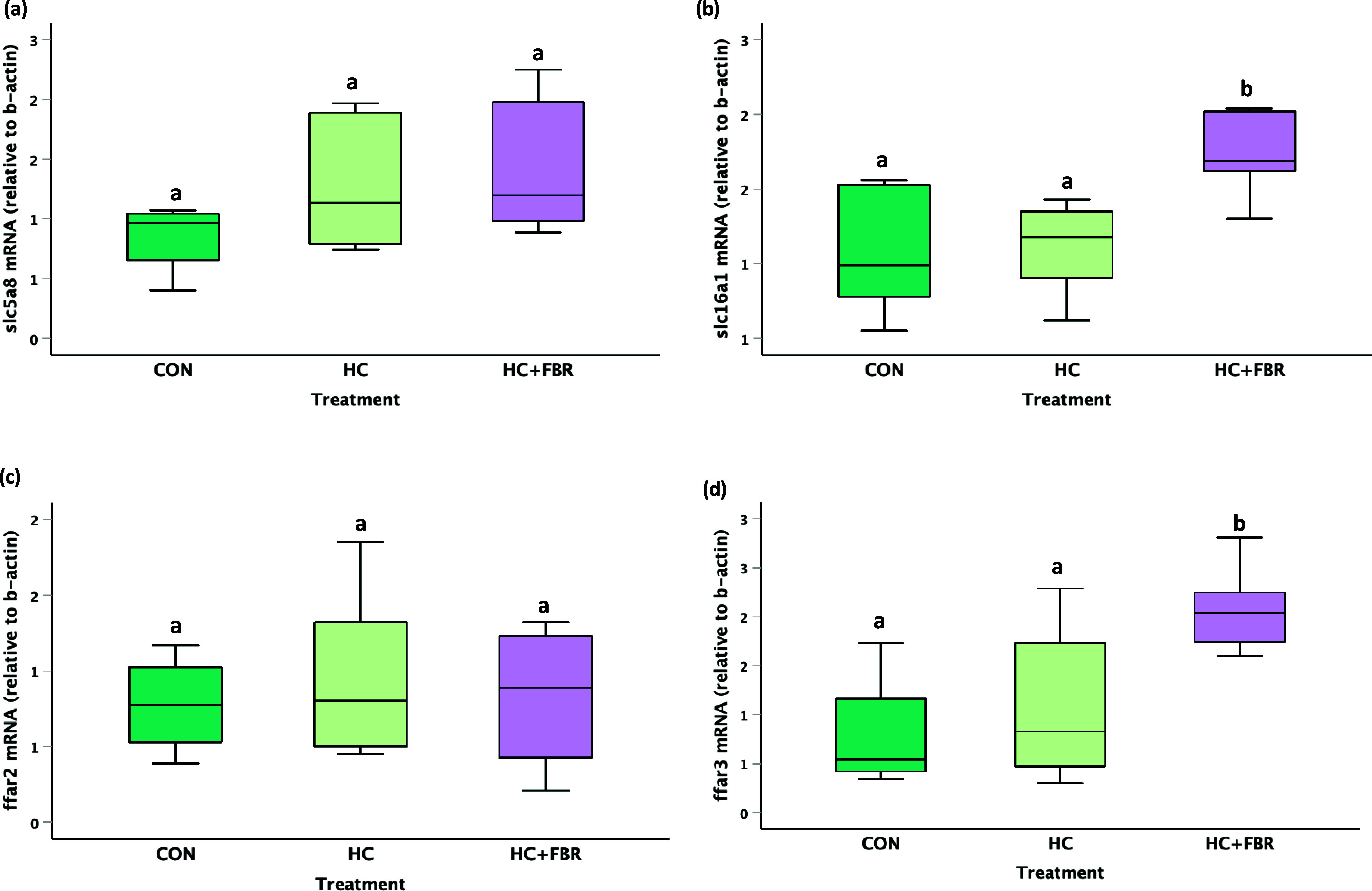
Maternal mRNA expression of SCFA transporters and receptors in caecal tissue on lactation day 21 including **(a)** slc5a8 (solute carrier family 5 member 8), **(b)** slc6a1(solute carrier family 6 member 1), **(c)** ffar2 (free fatty acid receptor 2), and **(d)** ffar3 (free fatty acid receptor 3). Data are presented as box and whisker plots, min to max, where the centre line represents the median. Data analysed using a one-way ANOVA with an LSD post-hoc multiple comparison test. **abc**, groups not sharing a letter are significantly different at *p* < 0.05; *n* = 6–8 animals per group.

No change (*p* > 0.05) was observed in slc5a8 (Figure 4a) or ffar2 (Figure 4c) mRNA expression between groups; however, mothers consuming the HC + FBR diet demonstrated increased (*p* < 0.05) mRNA expression of slc6a1 (1.6 fold of CON, Figure 4b) and ffar3 (2.6 fold of CON, Figure 4d) compared with CON and HC mothers.

### Offspring outcomes

#### Growth and serum biochemistry

**Figure 5.**
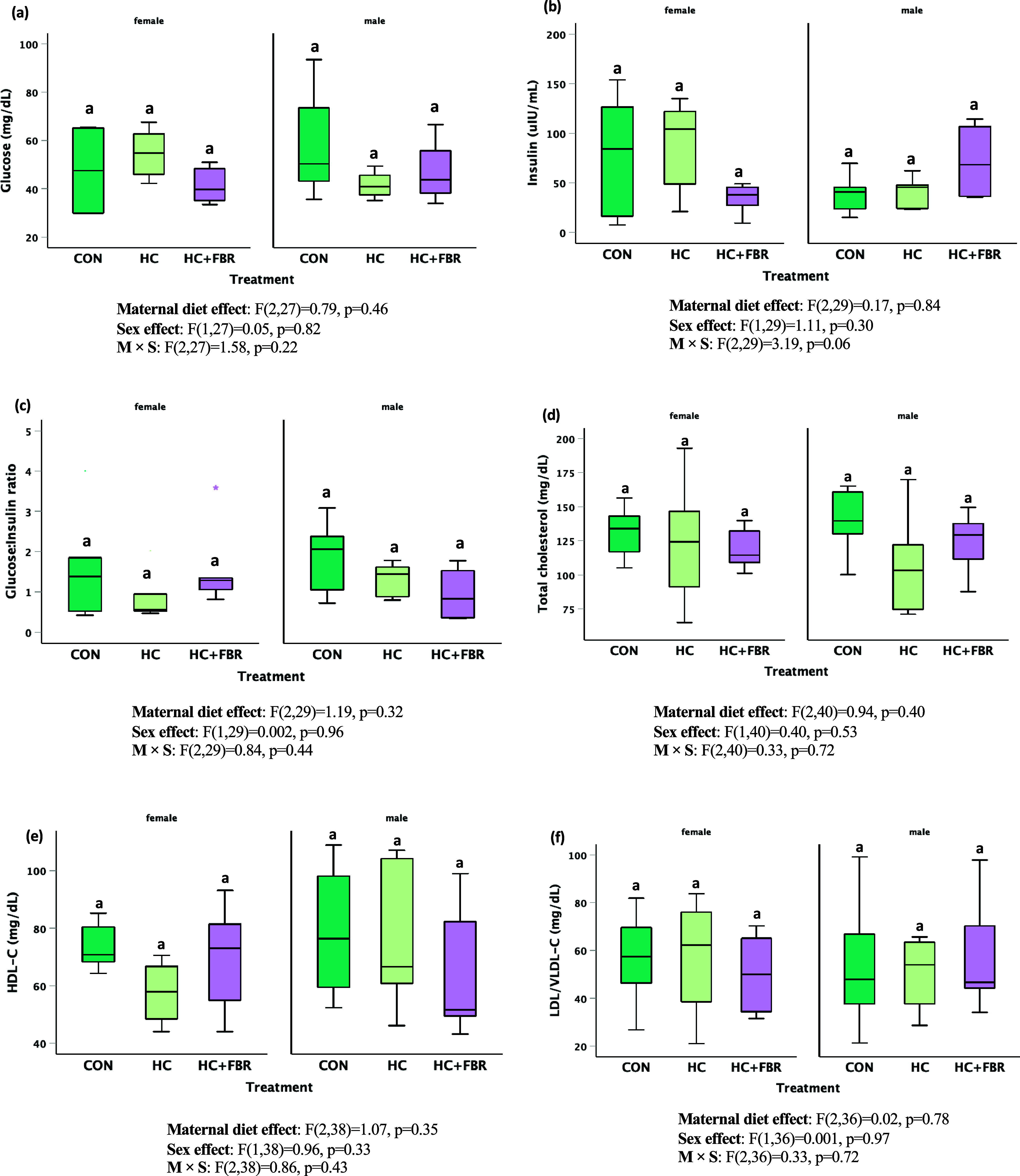
Serum biochemistry in newly weaned male and female pups including **(a)** Glucose (mg/dL), **(b)** Insulin (µIU/mL), **(c)** glucose:insulin ratio, **(d)** Serum total cholesterol (mg/dL), (**e**) Serum high density lipoprotein cholesterol (HDL-C, mg/dL), and (**f**) Serum low-density and very-low density lipoprotein cholesterol (LDL/VLDL-C, mg/dL). Data are presented as box and whisker plots, min to max, where the centre line represents the median. Data analysed using a one-way ANOVA with an LSD post-hoc multiple comparison test. **ab**, groups not sharing a letter are significantly different at *p* < 0.05; *n* = 6–8 animals per group.

Average pup weaning weight was higher (*p* < 0.05) in HC (69.3 ± 1.4 g) and HC + FBR (69.9 ± 2.5 g) groups compared with CON (56.5 ± 2.3 g). No differences were observed in serum glucose (Figure 5a, main effect, F_2,27_ = 0.79, *p* = 0.46; sex effect, F_1,27_ = 0.05, *p* = 0.82; M × S effect, F_2,27_ = 1.58, *p* = 0.22), insulin (Figure 5b, main effect F_2,29_ = 0.17, *p* = 0.84; sex effect, F_1,29_ = 1.11, *p* = 0.30; M×S effect, F_2,29_ = 3.19, *p* = 0.06) or the G:I ratio (Figure 5c, F_2,29_ = 1.19, *p* = 0.32; sex effect, F_1,29_ = 0.002, *p* = 0.96; M × S effect, F_2,29_ = 0.84, *p* = 0.44) in newly weaned male and female offspring. Similarly, no differences were observed in serum lipids including total-C (Figure 5d, main effect, F_2,40_ = 0.94, *p* = 0.40; sex effect F_1,40_ = 0.40, *p* = 0.53; M × S effect, F_2,40_ = 0.33, *p* = 0.72), HDL-C (Figure 5e, main effect, F_2,38_ = 1.07, *p* = 0.35; sex effect, F_1,38_ = 0.18, *p* = 0.96; M × S effect, F_2,38_ = 0.86, *p* = 0.43), or LDL/VLDL-C (Figure 5f, main effect, F_2,36_ = 0.02, *p* = 0.78; sex effect, F_1,36_ = 0.001, *p* = 0.97; M × S effect F_2,36_ = 0.33, *p* = 0.72) between treatment groups.

#### Liver lipids

**Figure 6.**
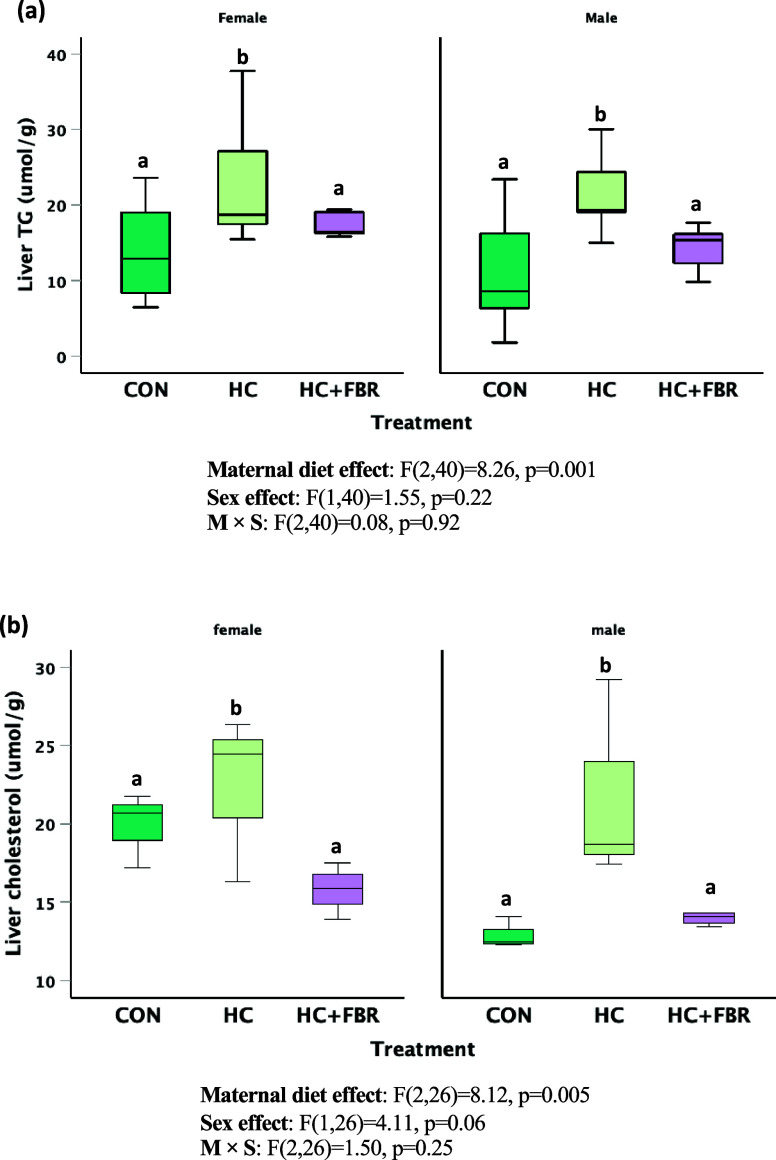
Hepatic lipids in newly weaned male and female pups including **(a)** Hepatic triglycerides (µmol/g) and (**b**) Hepatic cholesterol (umol/g). Data are presented as box and whisker plots, min to max, where the centre line represents the median. Data analysed using a one-way ANOVA with an LSD post-hoc multiple comparison test. **ab**, groups not sharing a letter are significantly different at *p* < 0.05; *n* = 5–8 animals per group.

Both male and female offspring from HC mothers demonstrated increased hepatic TG (vs. CON) that was normalised to CON levels in HC + FBR offspring (Figure 6a, main effect, F_2,40_ = 8.26, *p* = 0.001; sex effect, F_1,40_ = 1.55, *p* = 0.22; M × S effect, F_2,40_ = 0.08, *p* = 0.91). Similarly, male and female HC offspring had higher liver cholesterol which were lowered by the maternal HC + FBR intervention (Figure 6b, main effect, F_2, 26_ = 8.12, *p* = 0.005; sex effect, F_1,26_ = 4.11, *p* = 0.06; M × S effect, F_2,26_ = 1.50, *p* = 0.25).

#### Caecal SCFA

**Figure 7.**
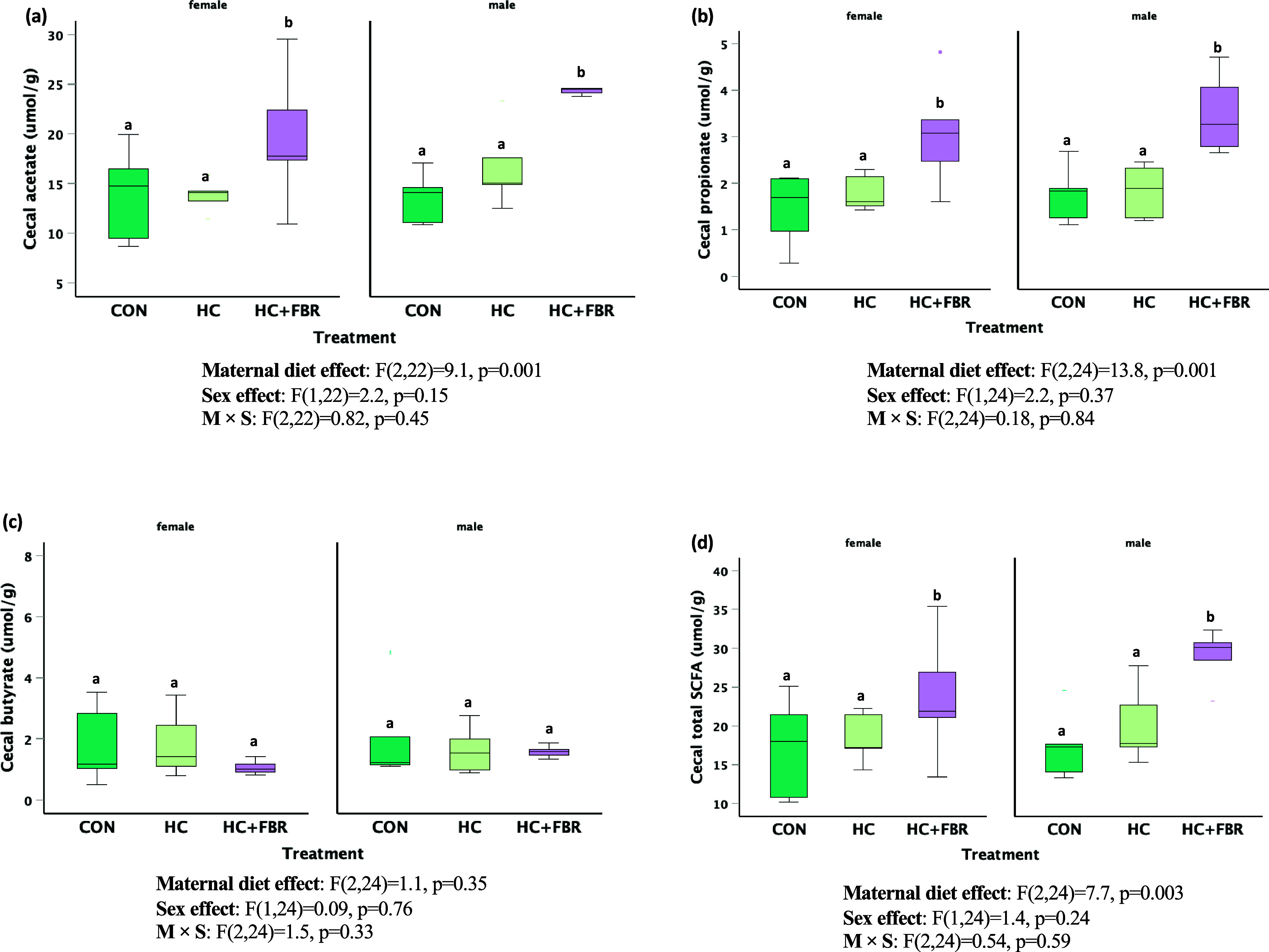
**(a)** Offspring short-chain fatty acid (SCFA) concentrations (µmol/g) in luminal caecal contents on postnatal day 21 including **(a)** Acetate, (**b**) Propionate; (**c**) Butyrate; and (**d**) Total caecal SCFA. Data are presented as box and whisker plots, min to max, where the centre line represents the median. Data analysed using a one-way ANOVA with an LSD post-hoc multiple comparison test. **ab**, groups not sharing a letter are significantly different at *p* < 0.05; *n* = 5–8 animals per group.

Caecal concentrations of acetate (Figure 7a, main effect, F_2, 22_ = 9.1, *p* = 0.001; sex effect, F_1,22_ = 2.2, *p* = 0.15; M × S effect, F_2,22_ = 0.82, *p* = 0.45), propionate (Figure 7b, main effect, F_2,24_ = 13.8, *p* = 0.001; sex effect, F_1,24_ = 2.2, *p* = 0.37; M × S effect, F_2,24_ = 0.18, *p* = 0.84), and total SCFA (Figure 7d, main effect, F_2,24_ = 7.7, *p* = 0.003; sex effect, F_1,24_ = 1.4, *p* = 0.24; M × S effect, F_2,24_ = 0.54, *p* = 0.59) were higher in HC + FBR offspring compared with newly weaned pups from the CON and HC groups. No difference in caecal butyrate concentrations were noted between groups (Figure 7c, main effect, F_2,24_ = 1.1, *p* = 0.35; sex effect, F_1,24_ = 0.09, *p* = 0.76; M × S effect, F_2,24_ = 1.5, *p* = 0.33).

## Discussion

This study assessed potential shifts in the maternal microbiome in response to yellow-pea prebiotic fibre supplementation in obese pregnancies and evaluated its protective role in mitigating liver steatosis in newly weaned offspring. Obese-complicated pregnancies were characterised by a reduction in beta diversity of the maternal microbiome and lower total caecal SCFA concentration. Maternal pea fibre supplementation (HC + FBR) differentially altered the caecal microbiome profile of several species, enhanced caecal SCFA concentration, and increased the mRNA expression of caecal targets involved in the carrier-mediated entry (slc6a1) and sensing (ffar3) of SCFA in the large intestine. We noted fibre-induced increases in the abundance of *Bifidobacterium pseudolongum*, several Provetella species (e.g., *Prevotella oris*, Prevotella copri, and *Prevotella ruminicola*), *Porphyromonas gingivalis*, and Paraprevotella xylaniphila and a lower abundance of Adlercreutzia equolifaciens, *Desulfovibrio piger*. Hepatic steatosis was evident in newly weaned offspring from mothers with obesity, whereas offspring from HC + FBR mothers demonstrated normalised liver lipid concentrations and an increase in caecal acetate and propionate concentrations.

Similar to our findings, previous animal model studies have a reported reduced within-(alpha) and between (beta) sample bacterial diversity in models of maternal obesity,^(57,58)^ although the differentially affected species are unique across studies and models. Interestingly, yellow pea fibre supplementation had little impact on diversity versus the HC + FBR diet. Although increased microbial diversity in response to whole pulse food intake has been observed,^(59)^ other animal model studies have reported variable and distinct differences in diversity outcomes depending on the type of pulse being fed (i.e., chickpeas, lentils, and bean)^(60,61)^ and the diversity index used.^(62)^ These differences may be driven by the unique physiochemical and nutritional properties of different pulses, all with specific fibre fractions with variable amounts and types of fibres as well as different levels of crude protein, starch, and fat.^(63)^

The dysbiosis observed in HC mothers (vs. CON) was also associated with reduced caecal SCFA concentrations, including both acetate and propionate. Fibre-supplemented mothers, on the other hand, demonstrated normalised concentrations of acetate and propionate, and an even higher concentration of butyrate (compared with both CON and HC). Further, caecal butyrate concentrations were positively associated with the abundance of 4 different species including *B. pseudolongum*, D. dubosii, *L. jensenii*, and *B. choerinum*. Previous maternal SCFA supplementation studies in rodent models suggest that placental and/or milk-mediated transfer of butyrate can prevent placental growth restriction,^(64)^ alter TG and cholesterol synthesis,^(65,66)^ and prevent hypertension in offspring.^(67)^ These findings, together with the observed caecal increases in the SCFA transporter SLC16a1^(68)^ and FFAR2, a G-protein-coupled transmembrane receptor that mediates the cellular effects of SCFAs,^(69)^ suggest that maternal fibre supplementation increased microbial fermentation activity and SCFA production, potentially mediating the improvement in liver steatosis in offspring.

Newly weaned male and female offspring from HC mothers demonstrated high liver TG and cholesterol concentrations compared with CON offspring. Although the long-term influence of this steatotic state is not known, excessive accumulation of both TG and cholesterol both contribute to the lipotoxicity that has been implicated in the pathogenesis of MASLD.^(70)^ Previous human maternal/child cohorts studies have also reported a link between maternal obesity and a higher risk and severity of MASLD in offspring.^(71,72)^ Mechanistic studies in animal models suggests that this association is mediated through multiple potential mechanisms including impaired autophagy response,^(73)^ increased lipid peroxidation,^(74)^ and epigenetic programming.^(9,75)^ Alternatively, offspring from HC + FBR-supplemented mothers demonstrated a normalised hepatic concentration of TG and cholesterol, highlighting a potential role for increased maternal fibre intake in protecting against the early-life programming of liver steatosis.

There is evidence that maternal high fat feeding during pregnancy and/or lactation may programme hepatic fat accumulation in offspring through alterations in the maternal microbiome, potentially shifting the composition of the microbial pool that is inherited by the offspring during early developmental periods.^(4,76,77)^ During gestation, the maternal gut microbiota can influence fetal metabolism through direct colonisation of fetal tissues (presumably following gut epithelium translocation by dendritic cells) and/or indirectly through the placental transfer of maternal bacterial-derived metabolites (i.e., amino acids, SCFA).^(14)^ Further, the maternal milk microbiome also influences the early life colonisation of infants.^(78)^ Schade et al. (2022)^(79)^ reported that the microbiota of Sprague-Dawley rat offspring at weaning was more influenced by the maternal lactation diet versus the pregnancy diet. Thus, it is conceivable that the observed pattern of liver fat accumulation in offspring at weaning may have been influenced by diet-induced modifications to the maternal microbiome. This hypothesis is strengthened by the lack of observed effects of the FBR treatment on maternal body weight and metabolic parameters. Further, although we did not evaluate the gut microbiome profile in offspring, the observed increase in caecal SCFA levels, particularly acetate and propionate, in HC + FBR offspring suggest a shift in bacterial composition and/or activity. In the mothers, however, fibre supplementation altered the abundance of several species that have been linked with liver fat metabolism and MASLD progression.

First, consumption of the HC + FBR diet shifted the maternal caecal microbiome toward a higher abundance of *B. pseudolongum*, a finding that has been reported in previous diet-induced obese mouse models supplemented with prebiotic fibre.^(80–82)^ Alternatively, supplementation of pulses, including chickpea flour^(83)^ and whole common beans^(84)^ has been reported to reduce the abundance of *B. pseudolongum* in both lean and obese mice. These conflicting findings suggest that shifts in microbial abundance may vary depending on the type of isolated dietary fibre or fibre-rich food source consumed.^(85)^ Nevertheless, probiotic supplementation studies with Bifidobacterium have noted species-specific improvements in MASLD through various mechanisms including reduced liver fat accumulation, improved hepatic inflammatory outcomes, and increased SCFA production.^(86–88)^ With respect to the latter, we noted higher caecal acetate concentrations in both HC + FBR mothers and their offspring, a main fermentation end-product of Bifidobacterium.^(89)^ Previous human work has reported an inverse association of between acetate and MASLD,^(90,91)^ likely through its regulatory role in reducing liver fat by inhibiting fatty acid synthesis and upregulating fat oxidation through AMPK and ERK1/2 signalling.^(92)^ Further, Song *et al*. (2023) reported that oral gavage of *B. pseudolongum* had a tumour suppressing effect that was mediated through acetate production in two mouse models of non-alcoholic fatty liver disease-associated hepatocellular carcinoma.^(93)^ Thus, increased acetate production, whether mediated directly through offspring fermentation activity or indirectly through maternal transfer may have contributed to the protective effect of FBR on hepatic steatosis.

Second, microbiota from fibre-supplemented dams were enriched in *P. gingivalis*, a species that has also been linked with hepatic steatosis.^(94)^ Although generally studied as an oral pathogen of chronic periodontitis,^(95)^ an oral-gut translocation of *P. gingivalis* has been demonstrated and linked with whole-body insulin resistance.^(96)^ *P.* gingivalis inoculation in diet-induced obese mice was associated with the progression of fatty liver disease.^(97)^ Alternatively, intravenous and oral administration of *P. gingivalis* in pregnant C57BL/6J dams elicited a down-regulation of TG synthesis-related genes and a numerical reduction in hepatic TG.^(98)^ Interestingly, a previous human prospective cohort study found that higher levels of *P. gingivalis* in saliva were linked to reduced fertility in women.^(99)^ Although we observed reduced reproductive success in HC dams that was normalised by FBR supplementation, this effect does not appear to be related to *P. gingivalis* as bacterial abundance was not different between HC and CON dams but was increased in the HC + FBR group. Nonetheless, a previous study does lend some support to a microbiota-mediated improvement in fertility following increased fibre consumption. Komiya *et al*. (2020) reported differences in gut microbial abundance between fertile and infertile women and increased pregnancy success linked with reduced dysbiosis in women supplemented with partially hydrolysed guar gum.^(100)^ Further, Tian et al. recently reported improved reproductive performance in response to dietary pea fibre supplementation in mice.^(101)^

Finally, we also noted fibre-induced increases in 12 different Prevotella species including P. copri. Prevotella species are abundant in both the oral and gut microbiome communities and have a high genomic diversity that likely explains both the beneficial and pathogenic health effects that have been reported.^(102)^ Prevotella species possess high SCFA production potential (particularly butyrate) through expression of carbohydrate active enzymes^(103,104)^ and have been shown to increase in abundance following consumption of fibre-rich diets.^(^ ^105^ ^–107)^ Previous work on the health effects of P. copri is variable, showing dual effects of 4 distinct sub-species.^(108)^ A higher abundance of P. copri has been associated with obesity,^(109,110)^ reduced glycaemic control,^(110,111)^ and MASLD.^(112–115)^ However, other studies have reported a causal improvement in glycaemic control from Prevotella copri administration.^(116,117)^ It has been suggested that this variability in metabolic strain-specific health responses is diet-dependent, with western diet-induced strains causing negative health effects and fibre-induced strains eliciting improved health responses.^(118,119)^ Indeed, diet-induced increase in P. copri following consumption of barley kernel-based bread mediated improved glycaemic control in healthy subjects.^(106)^ Nevertheless, although P. copri abundance was increased in the fibre-supplemented mothers in our study, we observed no impact on glycaemic control in mothers or offspring.

These findings suggest that maternal prebiotic fibre supplementation in obese pregnancies offers protection against hepatic lipid accumulation in newly weaned offspring. This protective response may be related to changes in the maternal microbiome profile as reflected by species composition changes and SCFA production patterns. Although microbiome composition was not directly measured in the offspring, changes in caecal SCFA concentrations may indicate a potential shift in the early microbiome colonisation which may have contributed to the observed lipid response in offspring of HC + FBR mothers.

## New and noteworthy

These findings suggest that maternal prebiotic fibre supplementation in obese pregnancies offers protection against hepatic lipid accumulation in newly weaned offspring, possibly by changing the maternal microbiome profile.
